# Endemicity change of hepatitis A infection necessitates vaccination in food handlers: An Indian perspective

**DOI:** 10.1080/21645515.2020.1868820

**Published:** 2021-02-17

**Authors:** Bhaskar Shenoy, Anar Andani, Shafi Kolhapure, Ashish Agrawal, Jaydeep Mazumdar

**Affiliations:** a Department of Paediatrics, Division of Pediatric Infectious Diseases, Manipal Hospital, Bangalore, India; b Global Medical Affairs, GSK, Belgium; c Medical Affairs Department, GSK, Mumbai, India; d Medical Affairs Team, GSK, Hyderabad, India; e Medical Affairs Team, GSK, Kolkata, India

**Keywords:** Hepatitis A virus, food safety, India, food handler, endemicity, outbreak, adolescent, adult, public health, vaccination, control measures

## Abstract

In the last two decades, outbreaks due to the foodborne hepatitis A virus (HAV) have been frequently reported in India, with adolescents and adults primarily affected. In India, most food handlers are adolescents and young adults who might be exposed to unsatisfactory environmental conditions and poor water quality. This increases the risk of HAV infection and consequently compounds the risk of HAV transmission from food handlers to susceptible populations. Given the shift in hepatitis A endemicity from high to intermediate levels in India, implementing the vaccination of food handlers has become important as it can also contribute to the elimination of hepatitis A in India. This narrative review makes a case for hepatitis A immunization of food handlers in India considering the growing food industry, evolving food culture, and the substantial burden caused by hepatitis A outbreaks.

## PLAIN LANGUAGE SUMMARY

What is the context?

- Hepatitis A disease is a common form of viral hepatitis and is transmitted through contaminated food and water or through close contact with an infected person. The virus with stands high temperature and can survive on surfaces for long periods of time.
- In India, the burden of hepatitis A has shifted from children to adolescents and adults who are more culnerable to infection. They present a high risk of complications, often requiring hopitalization.
- The prevention of the disease has often bee neglected, inadequate safety measures for the preparation of food (via food handlers) is a known risk factor for the transmission of hepatitis A.

What is new?

- Our review highlights the relationship between food handling and hepatitis A infection among adolescents and adults in Inida.
- The lack of knowledge of food safety regulations and hygiene measures among food handlers and the organizations that guide them may contribute to the spread of hepatitis A.

What is the impact?

- Sanitation efforts, awareness and educational programs for food are needed to help reduce the transmission of hepatitis A virus and disease, yet these measures alone may not be sufficient.
- Vaccination among high-risk populations such as food handlers can prevent hepatitis A infection and its complications as well as transmission.

## Introduction

Hepatitis A virus (HAV) causes infection through the fecal-oral route. It is usually asymptomatic in children as compared to higher age groups in whom complications such as acute liver failure, autoimmune hepatitis, and others have been reported.^1^ Typically, clinical illness resolves within two months, with approximately 10%–15% of persons showing prolonged or relapsing symptoms for up to 6 months^1,2^ and death in rare situations.^3^

Hepatitis A is estimated to cause over 100 million cases and around 15,000–30,000 deaths per year worldwide.^4^ It occurs intermittently and is known to cause outbreaks, with a trend of cyclic recurrences.^1^ Globally, food contaminated with HAV accounts for 2–7% of all HAV outbreaks.^5^ Infected individuals may require hospitalization which places a large strain on individual households expenses.^6^ According to global estimates, approximately 600 million cases of foodborne illness related to 31 pathogens are reported annually, of which 14 million cases are due to HAV infection with 1,353,767 Disability-Adjusted Life-Years (DALYs),^7^ which reflects the ability of the virus to persist in the environment and withstand food-production processes.^4^ Food handlers play a major role in preparation of safe food, and they may transmit HAV to susceptible individuals, if infected themselves.^2,8,9^ In India, lack of knowledge and awareness of preventing foodborne diseases and practice of proper hygiene among food handlers is a major concern as it may lead to a significant disease burden due to foodborne infections such as hepatitis A.^10^

To date, there is no overview of the HAV disease burden in the context of food handling in the Indian setting. Thus, the objective of this review is to summarize evidence from published literature in order to create awareness among healthcare providers, the scientific community, and policymakers on the role of food handlers in HAV transmission and present strategies to prevent, control, and eliminate hepatitis A disease and related outbreaks in India (see Plain language summary).

## Hepatitis A in adolescents and adults: a shift in disease endemicity

In regions where sanitary conditions have improved rapidly, cases of HAV in adolescents and adults are accumulating as they do not have immunity resulting from exposure in early childhood.^11^ In India, the burden of HAV infection has been on the rise with the disease being primarily reported in adolescents and adults.^12^ In a community study of hepatitis A in India, it was observed that the attack rate was highest among the 15–24 years age group (4.6%) followed by 3.1% and 1.2% in 5–14 and <5 years age groups, respectively.^13^ Attack rate for individuals >25 years of age was lower than 1%.^13^ Although milder illness in children might not have been captured and no statistical analysis was conducted in the study, attack rates of this magnitude reflect the vulnerability of the adolescent and adult population to HAV.^13^ According to recent census data, India has a population of roughly 1.38 billion people, of which approximately 63.7% are 15–59 years of age and economically active.^14^ Given the attack rates in adolescents and adults, and the changing social and cultural behaviors in India,^15^ it is a reasonable assumption that with the shifting HAV endemicity in India,^12^ HAV infection could have a large impact on individual health, and families due to loss of income and productivity.

## Current status of the burgeoning food industry in India

In recent years, India has undergone rapid socioeconomic growth resulting from increasing trade and industry, increasing degree of modernization, and access to better education, health services, housing, nutrition, and technology. This has led to an increase in disposable income which is leading to an unprecedented change in consumer behavior. Coupled with favorable demographics, this has led to a rapid growth in the food sector. The Indian food industry is projected to grow at a compounded annual growth rate of approximately 10% per year (2020–2025)^16^ with multinational fast food corporations being the major segments.^17^ Furthermore, according to a statistical report, the number of street food stalls in India are also steadily growing with an increase from 920,000 in 2008 to 1.2 million in 2013.^18^ These data are indicative of the increasing numbers of people opting to eat out at food establishments.

## Evolution of food culture in India

**Figure 1.**
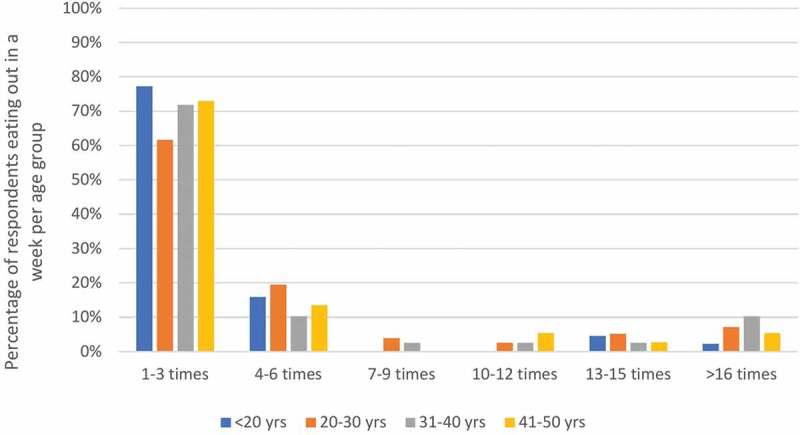
Habits of eating outside in India (number of times per week) per age group. Source: Adapted from Srividhya, 2014.^19^yrs, years

Given the socioeconomic progress in India, there has been a transition in population behavior, attributable mainly to the steadily rising income levels of the Indian population. The spending capacity of the Indian working population between 25 and 49 years of age is amongst the top three in the world.^17^ Given that the majority of the population is active in the workforce, this has led to an increase in overall spending and consequently to a change in eating out habits. Figure 1 shows that among individuals below 50 years of age, about 70% would dine outside 1–3 times per week, and 15% would eat out 4–6 times per week; among individuals of 20–30 and 31–40 years of age, up to 10% would eat out more than 16 times in one week. Notably, 89% of individuals who ate outside of their home indicated that cleanliness was important or extremely important.^19^ Reasons for eating outside of home are related to time constraints of individuals which are driven by both men and women increased focus on their career.^17^ Adolescents and adults in India have a culture of eating and socializing at street food stalls, attending weddings and other social events which are found ubiquitously across the country. Also, the number of educational institutes according to the All India Survey on Higher Education (AISHE) in 2018–19, were approximately 993 universities, 39,931 colleges and 10,725 stand-alone institutions in India with cafeterias available in 87%, 57% and 61% of these institutions, respectively.^20^ A large proportion of the Indian population is also known to travel long distances for work or other reasons; between 2010 and 2019, the Indian railway network transported over 23 million passengers every day.^21^ Given the duration of these long journeys, passengers are compelled to obtain food provided by catering facilities or independent food retailers who operate on the trains.

**Figure:**
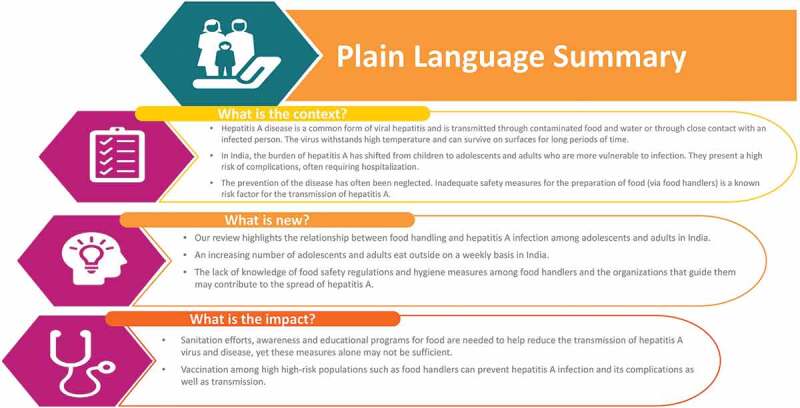

Collectively, these data reflect that a large population in India is potentially at risk of being exposed to foodborne HAV infection.

## Role of food handling in HAV outbreaks in India

According to the National Center for Disease Control (NCDC), an outbreak due to a foodborne disease is declared when two or more cases of a similar illness occur after the consumption of a common food or the observed number of cases of a specific disease exceeds the anticipated number.^22^ In India, incidence data for hepatitis A are limited or generally lacking, but despite the lack of surveillance system, few information on HAV incidence is nevertheless available, as reported through the Integrated Disease Surveillance Programme (IDSP) at NCDC.^23^ According to the NCDC, there were a total of 315 outbreaks of viral hepatitis between 2010 and 2013, and HAV was responsible for 10%–30% of acute viral hepatitis cases and 5%–15% of acute liver failure cases in India.^23^

Table 1 provides a summary of publications describing HAV outbreaks reported across India among adolescents and adults covering the time period 2004–2020.^13,24–35^ A majority of the studies documented the source of the outbreaks as unsafe food handling, unhealthy personal hygiene, or unsafe sanitation practices.^24,25,27,29,30,32–35^ Outbreaks were also linked to the utilization of contaminated water or food by hotels and restaurants.^13,26,28^ For example, one study reported that an outbreak occurred due to the use of unclean water in a welcome drink at a wedding ceremony.^31^ Across studies, the adolescent and adult groups were reported to have the highest number of cases (Table 1). This observation is clearly noticeable in regions with better sanitation, hygiene, and higher standards of living.^13,26,28–32,34^ While there were a few reports of cases in children,^25,33,35^ most cases in other states were also reported in adolescents and adults (Table 1).

**Table 1.** HAV outbreaks among adolescents and adults in India

Reference	Year of study	Location	No. of HAV cases	Diagnosis	Age group (years)	Source of infection
Gurav et al., 2019^26^	2016	Ernakulam, Kerala	73	Anti HAV- IgM	20–39*	Private well of restaurant
Kurup et al., 2019^28^	2016	Ernakulam, Kerala	236	Anti HAV-IgM	16–30*	Hotel drinks and water
Rakesh et al., 2018^13^	2016	Ernakulam, Kerala	142	Anti HAV-IgM	16–30*	Food from newly opened hotel
Kadri et al., 2018^27^	2015–17	Kashmir	24	Anti HAV-IgM	12–31*	Contaminated water supply
Rakesh et al., 2017^34^	2012–2016	Multiple districts, Kerala	2012: 14 outbreaks (1 with >50 cases, 7 with >20 cases) 2013: 19 outbreaks (3 with >50 cases, 12 with >20 cases) 2014: 18 outbreaks (1 with >100 cases, 4 with >50 cases, 9 with >20 cases) 2015: 20 outbreaks (3 with >50 cases, 11 with >20 cases) 2016: 13 outbreaks (1 with >100 cases, 2 with >50 cases, 10 with >20 cases)	Anti HAV-IgM	nr (adolescents and adults)	Contaminated water supply (at least 2 outbreaks)
Zachariah et al., 2017^31^	2016	Palakkad, Kerala	18	Anti HAV-IgM	15–25*	Welcome drink in marriage ceremony
Raveendran et al., 2016^30^	2015	Kollam, Kerala	98	Not mentioned	26–35*	Contaminated water supply
Rakesh et al., 2014^29^	2013	Kollam, Kerala	22	Anti HAV-IgM	15–24*	Contaminated water supply
Arora et al., 2013^24^	2011	Bhatinda, Punjab	9	Anti HAV-IgM	<20*->20	Contaminated water supply
Chobe and Arankalle, 2009^25^	2007	Shimla, Himachal Pradesh	38	Anti HAV-IgM	11–25*	Contaminated water supply
Chadha et al., 2009^33^	2004	Pune, Maharashtra	179	Anti HAV-IgM	0–15 (5–10*)	Contaminated water supply
Arankalle et al., 2006^32^	2004	Kottayam, Kerala	73	Anti HAV-IgM	18–52 (18–35*)	Contaminated water supply
Srinivasan et al., 2020^35^	2019	Vellore, Tamil Nadu	18	Anti HAV- IgM	nr (children)	Contaminated water supply

*Highest cases documented in the reported age groups

nr, not reported; HAV: hepatitis A virus; IgM: immunoglobulin M

## Lessons learned from foodborne HAV outbreaks

Given India’s socioeconomic diversity, there is a significant variation in the occurrence of HAV outbreaks within India; importantly, several lessons can be learned. While it is important that the risk of outbreaks in different regions should be closely monitored and proactively mitigated, strategies to prevent HAV infection and transmission are urgently needed in the country. In this context, the phrase “From farm to plate, make food safe”^36^ for food safety might be useful, as it could help in developing a preventive strategy composed of different complementary measures such as improving sanitation and access to clean drinking water, maintaining healthy food and water hygiene practices, and immunization.^4^

## Barriers and challenges for food handlers

It is essential to understand the barriers and challenges faced by food handlers to ensure the successful implementation of an intervention strategy to control, and ideally contribute to the elimination of hepatitis A in India. The main barrier that food handlers face whilst realizing the importance of food safety, is the lack of time to collect relevant regulatory information for their establishment,^37^ and assimilate the guidelines put in place by several bodies in India,^38^ like the Food Safety and Standards Authority of India (FSSAI).^39^ This is in part due to the complex regulatory framework that oversees the implementation of food safety principles.^9,38^ Furthermore, the dissemination practices related to updates on changes in regulations and guidelines of food safety adopted by these bodies are inadequate.^38^ Regarding small- and medium-scale food industries, Food Business Operators (FBO) have stated that they find it difficult to stay updated on relevant procedural and compliance changes and do not have the capacity to track regulatory changes.^38^ Even if food safety principles are implemented, relevant institutional and human resources to monitor food safety coupled with satisfactory laboratory and testing-capacity are generally unavailable in India.^38^

## Knowledge, attitudes, and practices among food handlers in India

The complexities related to food regulations, poor knowledge, general awareness about hygiene, and the improper safety practices of food handlers are known to impede the prevention of foodborne illnesses such as hepatitis A. Importantly, the lack of education or training influences the attitude of food handlers toward implementing food safety principles.^40–43^ A cross-sectional study among street food vendors revealed that a majority of them were familiar with the terms “food hygiene” and “foodborne illness.” However, less than one-third of these street food vendors had acceptable food preparation practices.^43^ An indifferent attitude toward implementing and maintaining food hygiene was significantly associated with age, gender, education, marital status and type of food vendor.^43^ Singh et al. showed that basic hygiene and sanitation practices were documented as less than satisfactory among food vendors due to a lack of adequate infrastructure and training related to food hygiene.^44^ Other studies have found similar associations.^40–42^ Collectively, this evidence suggests a high prevalence of general awareness about hygiene and food safety principles among food handlers, yet these principles were seldom implemented, mainly attributable to an indifferent attitude toward food safety.

## Strategies for the control and elimination of foodborne hepatitis A

In terms of strategies to minimize the risk of HAV outbreaks, several non-pharmaceutical and pharmaceutical interventions should be utilized to tackle HAV transmission due to food handling. First, in terms of non-pharmaceutical interventions, water quality should be improved and monitored regularly to ensure the provision of clean and safe drinking water for food handling and preparation. Second, chlorination of wells and the use of boiled water should be implemented for food, drink, and ice preparations. Finally, within the context of food preparation, food safety principles should be implemented in food establishments such as street food stalls, cafeterias, restaurants, and hotels. The importance of proper handwashing should also be stressed. In addition to these interventions, the spread of hepatitis A may be controlled and prevented through hepatitis A vaccination.

The World Health Organization (WHO) states that vaccination against hepatitis A should be part of a comprehensive plan for the prevention and control of viral hepatitis, including measures to improve hygiene and sanitation and measures for outbreak control. The WHO recommends implementation of HAV vaccination of children ≥1 years of age in universal vaccination programs of countries where a shift in endemicity has been documented, following epidemiology and cost-effectiveness considerations.^3,4,45^ Moreover, the WHO considers food handlers as a risk group.^4^

In India, to date, no universal vaccination recommendations are made for groups most susceptible to HAV, such as adolescents and adults or food handlers.^46^ However, the Indian Medical Association (IMA) and the Association of Physicians of India (API) recommend hepatitis A vaccination in special or high-risk individuals such as adolescents and adults,^47^ and food handlers.^48^ While vaccination of food handlers does not address the situation wherein food itself may be contaminated with hepatitis A, this strategy would be an important step in preventing the transmission of HAV to susceptible individuals.

## Hepatitis A vaccination of food handlers

Worldwide, there are several hepatitis A vaccines available for use in adolescents and adults; clinical trials have reported that hepatitis A vaccination is safe and efficacious in these populations.^3,4^ The remarkable immunogenicity of hepatitis A vaccines in adolescents and adults is reflected in rapid seroconversion rates, enabling both preexposure and postexposure prophylaxis.^3,4,49^ The efficacy and safety of hepatitis A vaccination have been established in children, adolescents, and adults.^3,4^

The value of immunization of food handlers has also been demonstrated in the real-world setting, as in the United States (US). Despite a low HAV endemicity, several hepatitis A outbreaks in St. Louis (two restaurants and one cafeteria) led to the implementation of mandatory hepatitis A vaccination of food handlers. Consequently, the case rate of hepatitis A dropped from 3 per 100,000 population during the pre-hepatitis A vaccination period to 1 per 100,000 population, thereby lowering the morbidity and economic burden.^50^ Recently (2019), The Alabama Department of Public Health, Immunization Division, recommended hepatitis A vaccination for all food workers across the state, given that there were 132 confirmed outbreak-related cases in at least 25 Alabama counties.^51^ Such initiatives testify to the value of vaccination of food handlers in the prevention of hepatitis A and underscores the importance of local outbreak data in vaccine-policy decision-making. This is in line with recommendations from US Advisory Committee on Immunization Practices (ACIP) to vaccinate food handlers with hepatitis A vaccine based only on local epidemiological data.^52^ It is worth noting that mandatory hepatitis A vaccination for food handlers is not recommended by the US ACIP, probably because investigation reports of HAV infected food handlers transmitting infections in restaurants are not directly reported to the US Centers for Disease Control and Prevention (CDC) but reported in local newspapers.^53^ However, the ACIP recommendation state that contamination of food with HAV can occur from the time of harvest, processing, handling, or after cooking the food; and also in imported food items.^52^ In this context, the ACIP does recommend HAV vaccination for individuals requesting protection without acknowledgment of a risk factor.^52^ Few countries that have intermediate HAV endemicity in the Asia-Pacific region (e.g. Sri Lanka^54^) recommend the vaccination of high-risk groups such as food handlers. In Ireland, a country with low endemicity, recent guidelines stipulate that hepatitis A vaccination is recommended for food handlers who are not immune.^55^ Yet, to our knowledge, there is no experience of this strategy from countries that are experiencing a shift in HAV endemicity, like India.

In the Indian setting, HAV immunization of adults is often neglected or there is a lack of drive among policymakers, HCPs and other stakeholders. Moreover, low perception or access to vaccination and cost issues may also play an important role.^56,57^ A recent review on economic evaluations of hepatitis A vaccination strategies suggests that hepatitis A vaccination has favorable cost-effectiveness in middle-income countries if they are of high and intermediate endemicity.^58^ Given the shift from high to intermediate endemicity and the substantial cost of treating one HAV case (USD 346) in India,^6^ vaccination of food handlers to prevent the spread of HAV across the country could potentially be cost-effective, and even cost-saving when both direct and indirect costs of disease are taken into account. These reports should be a wake-up call for the policymakers to make hepatitis A vaccination mandatory for food handlers and not merely to rely on standard interventions such as investigation, education, proper sanitation, and hygiene.

## Discussion

This review article makes a case for the immunization of food handlers in India considering the growing food industry, evolving food culture, and hepatitis A outbreaks. We identified several outbreaks that occurred between 2004 and 2020 across several states in India which resulted from improper food handling practices and the use of inadequate water supply.^13,24–35^ Observations from outbreaks support a shifting endemicity in India with adolescents and adults being primarily affected by HAV infection.^13,24–35^ This finding is consistent with reports from other countries that have also seen improvements in socioeconomic indicators; with increasing incomes and improvements in access to clean water and adequate sanitation.^59,60^

In line with the 2030 WHO elimination targets for viral hepatitis,^61^ the Government of India initiated the National Viral Hepatitis Control Programme, the Swachh Bharat Mission campaign and the National Rural Drinking Water Programme (NRDWP) with an overall objective to raise awareness, reduce morbidity and mortality and provide safe, potable water for all.^62–64^ Moreover, regulations by the FSSAI^39^ and the Bureau of Indian Standards are available and are continually updated.^65^ It has become important to ensure that awareness and training programs about regulations related to procedural changes are optimized and communicated to food handlers in a timely manner. Within this context, identifying potential gaps in the knowledge, attitudes, and self-reported practices of food handlers is imperative to refine interventional strategies to promote food safety.

Using the recently published review on the changing HAV epidemiology in India^12^ with our findings, the evidence could be useful for healthcare providers and policymakers in India to help define hepatitis A prevention strategies, including targeted immunization of risk groups, such as food handlers. For middle-income nations, like India, resource constraints pose an important barrier to provision of and access to medical services such as immunization. Data on the levels of morbidity, comorbidity, and mortality due to hepatitis A are also needed to assess the size of the burden to healthcare systems and society.

The findings of this review must be interpreted with caution due to several limitations. First, reports of outbreaks from different regions are characterized by sociocultural and economic diversity. Second, epidemiological data from the outbreaks were not analyzed to assess whether hygiene and sanitation were indeed the reasons influencing the shift in average age of individuals infected with HAV in the different regions of the country. Despite these limitations, it is noteworthy to mention that most HAV outbreaks in India demonstrate that adolescents and adults were the most common population segment affected by the disease, which testifies to the shifting endemicity in India. This underscores the importance of vaccinating food handlers in India, the majority of whom are themselves adolescents and young adults.

## Conclusion

The burden of HAV outbreaks due to improper food handling procedures is substantial and growing in India, with food handlers being primarily exposed to the disease, and transmitting the disease to susceptible individuals. While the current paper focuses on the Indian setting, the principles may be relevant to other countries globally that showcase similar dynamics such as a shifting HAV endemicity (from high to intermediate), improving socio-economic conditions and an increase in overall disposable income to eat outside of the home. In this context, guidelines on the prevention and management of hepatitis A may need to be revised to be more comprehensive in their inclusion of risk groups. Governments of countries should view vaccination as an investment into their preventive health care programs, which would ultimately save on health care spend. Additionally, HCPs need to enhance the standard of care of adults and routinely offer vaccination to eligible individuals.

## Clinical Trial Registration

NCT Number (www.clinicaltrials.gov) and NA to come.
